# Allergic Diseases in Common Variable Immunodeficiency: A Prospective Cross‐Sectional Study on Prevalence and Allergy Biomarkers of Clinical Phenotypes

**DOI:** 10.1111/sji.70108

**Published:** 2026-03-17

**Authors:** Maria Giovanna Danieli, Ilaria Claudi, Stefania Auria, Matteo Martini, Elena Buti, Silvia Brunetto, Gianluca Moroncini, Paola Lucia Minciullo, Sebastiano Gangemi, Maria Beatrice Bilò

**Affiliations:** ^1^ SOS Immunologia delle Malattie Rare e dei Trapianti Ancona Italy; ^2^ SOD Clinica Medica, Dipartimento di Medicina Interna, Azienda Ospedaliero Universitaria delle Marche, and DISCLIMO Università Politecnica delle Marche Ancona Italy; ^3^ Postgraduate School of Allergy and Clinical Immunology Università Politecnica delle Marche Ancona Italy; ^4^ SOSD Allergologia, Dipartimento di Medicina Interna, Azienda Ospedaliero Universitaria delle Marche, and DISCLIMO Università Politecnica delle Marche Ancona Italy; ^5^ School of Allergy and Clinical Immunology University of Messina Messina Italy; ^6^ Operative Unit of Allergy and Clinical Immunology, Department of Clinical and Experimental Medicine University of Messina Messina Italy

**Keywords:** allergic rhinitis, allergy, asthma, common variable immunodeficiency, drug hypersensitivity reactions, IgE, immune dysregulation, inborn errors of immunity, intravenous immunoglobulin, subcutaneous immunoglobulin

## Abstract

The link between allergic conditions and common variable immunodeficiency (CVID) is still unclear. Only a few studies suggest allergic diseases are more prevalent in CVID patients than in the general population, and the role of IgE remains poorly defined. This study aims to evaluate the prevalence of allergic conditions in CVID and the role of serum IgE and IgA levels. This prospective, cross‐sectional, case–control study enrolled Italian adult CVID patients to investigate allergic conditions' frequency and relationships between IgE, IgA, and clinical phenotypes. Analyses of diagnostic/prognostic accuracy were performed with ROC curves. We documented an allergic disease in 26.6% of 60 CVID patients, most commonly allergic rhinitis (56.2%) and bronchial asthma (12.5%). CVID patients with allergy had higher IgE levels (+8.9 kU/L, *p* = 0.006) than non‐allergic ones, but lower than allergic individuals without CVID. IgE deficiency was observed in 65% of CVID patients, with a strong correlation between IgE and IgA levels (*r* = 0.7, *p* < 0.001). Low IgE (< 2.5 kU/L) and IgA levels (< 7 mg/dL) were significantly associated with lymphoproliferative (*p* < 0.001) and granulomatous phenotypes (*p* = 0.005), achieving an AUC of 94% and 81% for predicting lymphoproliferation and granulomatosis, respectively. The prevalence of allergic conditions in CVID patients is lower compared with previous studies. Low IgE levels served as a good biomarker for CVID and CVID‐phenotypes. Combined serum IgE and IgA assessment improved prognostic stratification.

## Graphical abstract

Allergy occurred in 27% of 60 CVID patients, while IgE deficiency was observed in 65% and was strongly correlated with serum IgA levels. Low serum IgE/IgA levels accurately identified lymphoproliferative and granulomatous phenotypes, thereby improving diagnostic and prognostic stratification in CVID.

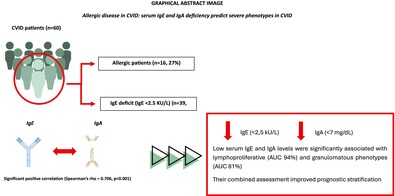

**Abbreviations**
AUC: area under the curve
CVID: Common variable immunodeficiency
DHR: drug hypersensitivity reactions
fSCIg: facilitated subcutaneous immunoglobulin
GINA: global initiative for asthma
IEI: Inborn Errors of Immunity
Ig: immunoglobulin
IgA: immunoglobulin A
IgE: immunoglobulin E
IgM: immunoglobulin M
IgG: immunoglobulin G
IVIg: intravenous immunoglobulin
IgRT: immunoglobulin replacement therapy
MDM: minor determinant
NSAIDs: non‐steroidal anti‐inflammatory drugs
PPL: major determinant
PRISMA: preferred reporting items for systematic reviews and meta‐analyses
ROC: receiver operating characteristic
SCIg: subcutaneous immunoglobulin
SIgAD: selective IgA deficiency
SPT: skin prick tests

## 1 Introduction

Common variable immunodeficiency (CVID) is the most common symptomatic inborn errors of immunity (IEI), characterized by defective immunoglobulin (Ig) production associated with impaired B cell differentiation [1]. Patients may present with heterogeneous comorbidities including recurrent bacterial infections, autoimmunity, gastrointestinal diseases, chronic pulmonary disease, polyclonal lymphoproliferation, and increased risk of malignancy [2, 3].

Scarce data are available about the prevalence of allergic conditions in patients with CVID [4, 5]. Previous studies have reported the prevalence of allergic diseases in CVID patients to be 12%–42%, varying widely across studies, including asthma, allergic rhinitis, food allergy, atopic dermatitis, and drug hypersensitivity [4]. Some studies have shown that allergic manifestations are among the most frequent clinical presentations because of mucosal immune defects and immune dysregulation [5], and therefore may impact the quality of life of these patients. Additionally, chronic lung disease is a common issue in patients with CVID, leading to recurrent hospitalizations and significant morbidity [6]. Many patients with CVID have a clinical history suggestive of allergic respiratory disease. Therefore, assessing asthma and atopy in these patients can be challenging, as it may not be possible to differentiate whether respiratory symptoms are attributable to infectious complications or allergic disease.

The role of atopy in CVID patients has not been well established, such as the role of serum total IgE in patients with primary antibody deficiency with a history of allergy compatible with an IgE‐mediated reaction [7, 8, 9, 10]. Currently, total serum IgE levels are not part of the routine diagnostic assessment for patients with recurrent infections and suspected hypogammaglobulinemia, and serum IgE is not considered in establishing a diagnosis of CVID [1]. Lawrence et al. considered low or undetectable serum IgE as a distinctive feature of CVID by comparing the incidence of such levels in healthy controls and individuals with CVID [11]. However, a recent retrospective study has highlighted the diagnostic role of total IgE measurement in the work‐up of newly discovered primary hypogammaglobulinemia [2].

Furthermore, data of literature showed that IgE‐deficient patients (IgE < 2.5 kU/L) have a high prevalence of malignancy compared with non‐IgE‐deficient individuals [8]. This increased malignancy association may be at least partially due to the proposed IgE possible role in tumour surveillance [8].

According to the PRISMA (Preferred Reporting Items for Systematic Reviews and Meta‐Analyses) guidelines [12], we performed a comprehensive literature review to evaluate the extent to which this relationship had been previously explored in research.

Through a prospective study, we studied the relationship between CVID patients and allergic diseases such as asthma, allergic rhinitis, food allergy, venom allergy, and drug hypersensitivity. Furthermore, we investigated the role of total and specific serum IgE in the diagnosis and prognosis of CVID patients.

## 2 Methods

This prospective, cross‐sectional, case–control study enrolled Italian adult patients with CVID, followed up according to what we already published [13, 14]. Asthma was diagnosed according to Global initiative for asthma (GINA) criteria [15]. The primary analysis investigated the total IgE and the prevalence of allergic diseases, compared with the general population. In the secondary analyses, we investigated the role of serum IgE and IgA, their relationship with clinical phenotypes and other Ig classes, and their diagnostic/prognostic accuracy.

The Authors declare no use of any Artificial Intelligence Generated Content (AIGC) tools such as ChatGPT and others based on large language models (LLMs) used in developing any portion of their manuscript.

A complete description of methods is available in the online Data S1.

## 3 Results

### 3.1 Baseline Characteristics

We examined 60 adult patients consecutively enrolled from 2023 to 2024, 39 females (65%) with a mean age ± SD at the diagnosis of CVID of 42 ± 18 years. The main clinical and laboratory characteristics are shown in Table 1.

**TABLE 1:** Baseline characteristics of CVID patients (*n* = 60) with clinical phenotypes according to Chapel et al. [3].

Gender: Female	*n* (%)	39 (65%)
Current age	*Mean ± SD* ^*1*^ *(range)*	56 ± 16 (27–90)
Age at CVID diagnosis (years)	*Mean ± SD* ^*1*^ *(range)*	42 ± 18 (10–82)
Diagnostic delay (years)	*Mean ± SD* ^*1*^	11 ± 13
Therapeutic delay (years)	*Mean ± SD* ^*1*^	12 ± 14
Mean serum IgG levels at diagnosis (mg/dL)	*Median [IQR]* ^*2*^	368 [260, 485]
Mean serum IgA levels at diagnosis (mg/dL)	*Median [IQR]* ^*2*^	7 [6, 30]
Mean serum IgM levels at diagnosis (mg/dL)	*Median [IQR]* ^*2*^	23 [13, 44]
Mean serum IgE levels (kU/L)	*Median [IQR]* ^*2*^	1 [1, 6]
No other disease‐related complications (“infections only”)	*n (%)*	31 (52%)
Autoimmunity	*n (%)*	23 (38%)
Polyclonal lymphoproliferation	*n (%)*	27 (45%)
Granulomatosis	*n (%)*	14 (23%)
Malignancy	*n (%)*	23 (38%)
Enteropathy	*n* (%)	5 (8%)
Ig replacement therapy		56 (92%)
IVIg		21 (35%)
20% SCIg	*n* (%)	16 (25%)
Facilitated subcutaneous immunoglobulin (fSCIg) therapy		19 (32%)
Antibiotic prophylaxis	*n (%)*	4 (6%)
Deceased patients	*n (%)*	6 (9%)

Abbreviations: CVID, common variable immunodeficiency; IV, intravenous; IQR, interquartile range; SC, subcutaneous; SD, standard deviation.

Thirty‐one patients (51.6%) exhibited a clinical phenotype characterized by “infections only”, whereas the remaining patients (48.4%) had a phenotype with at least one of the disease's complications, such as autoimmunity (38.3%), polyclonal lymphoproliferation (45%), enteropathy (excluding gastrointestinal infectious or autoimmune diseases, and celiac disease) (8.3%), and neoplastic disease (38.3%). Among the 27 patients with a clinical phenotype characterized by lymphoproliferation, 14 had granulomatosis. We frequently observed overlapping phenotypes in the study population (Table 1).

### 3.2 Prevalence and Main Characteristics of Allergy and Hypersensitivity Reactions in CVID Patients

**FIGURE 1:**
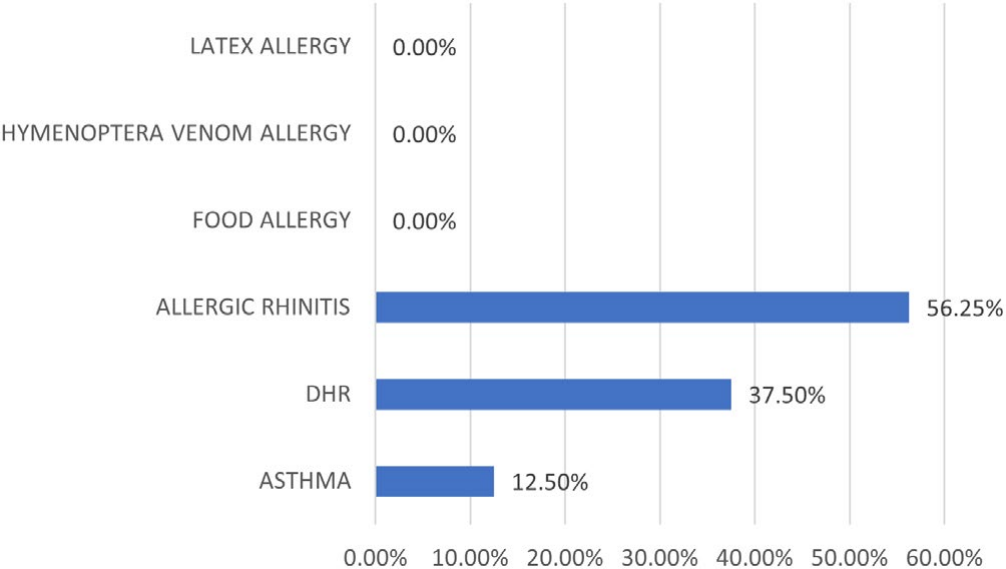
Frequency of allergy and hypersensitivity reactions in 16 CVID patients. DHR, Drug hypersensitivity reactions.

Of the 60 (26.6%) patients, 16 exhibited any kind of allergy and/or hypersensitivity reactions (Figure 1). Based on the classification reported in Table 2, 7/60 (11.6%) patients had confirmed respiratory IgE‐mediated allergy, characterized by allergic symptoms with positive skin prick tests (SPT) and/or specific IgE, and total IgE levels > 2.5 kU/L.

**TABLE 2:** Characteristics of 16 CVID patients diagnosed with allergy.

N. patients	Clinical	Inhalant skin prick tests	Total serum IgE levels
7	Allergic rhinitis	positive	> 2.5 kU/L in all patients
1	Allergic rhinitis	negative	< 2 kU/L
1	Allergic rhinitis and bronchial asthma	negative	< 0.01 kU/L
1	Bronchial asthma	negative	< 0.01 kU/L
2	Drug hypersensitivity reactions	n. a.	> 2.5 kU/L in both patients
4	Drug hypersensitivity reactions	n. a.	< 2 kU/L in all patients

Conversely, 3/60 patients (5%) reported allergic‐like respiratory symptoms without detectable sensitization, showing seasonal rhinitis or asthma‐like manifestations despite negative SPT results and low total IgE levels (< 2 kU/L) (Table 3). In particular, two patients exhibited allergic‐like rhinitis with negative skin tests and extremely low IgE (< 2 kU/L and < 0.01 kU/L, respectively), but due to the seasonal pattern and association with pollen exposure, an allergic aetiology was still suspected. Two patients exhibited asthma manifestations: one patient had asthma as the only allergic phenotype (with negative SPT and total IgE < 0.01 kU/L), whereas another presented with both asthma and allergic‐like rhinitis (negative SPT and very low IgE < 2 kU/L).

**TABLE 3:** Respiratory allergic phenotypes in CVID patients.

Group	Definition	n (%)	Diagnostic confirmation
Confirmed respiratory IgE‐mediated allergy	Allergic respiratory symptoms + positive SPT and/or specific IgE	7/60 (11.6%)	Positive SPT/sIgE, IgE > 2.5 kU/L in all
Allergic‐like respiratory symptoms without detectable IgE‐sensitization	Seasonal respiratory symptoms, with negative SPT and total IgE < 2 kU/L	3/60 (5%)	Negative SPT, low IgE

Abbreviation: SPT, Skin Prick Test.

Overall, six patients (6/60, 10%) reported a suspected drug hypersensitivity reaction (DHR) (Table 4). Five of them did not undergo any allergy work‐up (they refused SPT and drug provocation testing). One patient had a history of DHR and underwent testing, which yielded negative SPT (minor determinant and major penicillin determinants, amoxicillin–clavulanic acid) and negative specific IgE (penicilloyl G/V, ampicillin, cefaclor), leaving the IgE‐pathogenesis unconfirmed; she declined the drug provocation test. They reported reactions to beta‐lactams (*n* = 3), quinolones (*n* = 1), non‐steroidal anti‐inflammatory drugs (NSAIDs) (metamizole, *n* = 1), and intravenous immunoglobulin (*n* = 1). All reactions involved only cutaneous manifestations (angioedema, pruritus, or urticaria).

**TABLE 4:** Drug hypersensitivity reactions in CVID patients.

Group	Definition	*n* (%)	Diagnostic confirmation
Suspected DHR	History suggestive of Type B DHR involving beta‐lactams (*n* = 3), quinolones (*n* = 1), NSAIDs (metamizole, *n* = 1), or intravenous immunoglobulin (*n* = 1); no complete allergy work‐up	5/60 (8.3%)	No ST performed/no drug challenge
Evaluated DHR, negative testing	Drug reaction history with cutaneous manifestations only (angioedema, pruritus, urticaria); full testing performed for one patient	1/60 (1.6%)	Negative ST to MD, PPL, and amoxicillin–clavulanic acid; negative sIgE to penicilloyl G/V, ampicillin, and cefaclor; no oral provocation test

Abbreviations: DHR, drug hypersensitivity reaction; MD, minor determinant of penicillin; NSAIDs, non‐steroidal anti‐inflammatory drugs; PPL, penicilloyl polylysine as major determinant of penicillin; SPT, skin prick test.

In our series, patients with CVID and allergic/hypersensitivity reactions had statistically significantly higher IgE levels compared with other CVID patients without allergy (+8.9 kU/L, 95% CI 2.6–15.1, *p* = 0.006). A trend towards higher IgE serum levels was specific to CVID patients with pollen and dust mite allergies, compared with patients with DHR (+13.2 kU/L, 95% CI –4.3 to 30.6, *p* = 0.128). No cases of food, venom, or latex allergy were identified.

### 3.3 Serum IgE and IgA Levels in CVID Patients

Globally, 19/60 (31.6%) patients had total IgE serum levels exceeding the cut‐off of 2.5 kU/L, with an average IgE level of 18.5 kU/L. Among the 60 CVID patients, 39/60 (65%) had total IgE levels < 2.5 kU/L (IgE‐deficient patients). Total lgE levels could not be obtained in two patients who died during the follow‐up. Additionally, 6 of the 39 IgE‐deficient patients were allergic.

In our series, 27 (45%) patients had serum IgA levels > 7 mg/dL and < 40 mg/dL, with a mean of 36 mg/dL. Notably, 32/39 (82%) IgE‐deficient patients had IgA levels < 7 mg/dL. Thus, there was a statistically significant positive correlation between serum IgE and IgA values in our cohort (Spearman's rho = 0.706, 95% CI: 0.542 to 0.818, p = < 0.001).

### 3.4 Serum IgE Levels According to Clinical Phenotypes in CVID Patients

Table 5 shows the serum IgE and IgA levels according to the CVID clinical phenotype. All the 27 patients with lymphoproliferation had IgE levels < 2.5 kU/L (*p* < 0.001) with 82% of them also showing IgA levels < 7 mg/dL (*p* < 0.001) (Table 5). Additionally, 74% of our patients with an autoimmune phenotype had total IgE levels < 2.5 kU/L (*p* = 0.887), whereas 54% of patients with CVID who developed neoplasia had total IgE values < 2.5 kU/L (*p* = 0.852).

**TABLE 5:** Correlation among serum IgE and IgA levels and clinical phenotype according to Chapel [3].

Clinical phenotype	No. Patients	No. patients with IgE < 2.5 kU/L	No. patients with IgA < 7 mg/dL
Autoimmunity	23	17/23 (74%)	14/23 (61%)
Lymphoproliferation	27	27/27 (100%) *	22/27 (100%) *
Granulomatosis	14	14/14 (100%) *	12/14 (86%) *
Malignancy	26	14/26 (54%)	12/26 (46%)
All CVID patients	60	39/60 (65%)	27/60 (45%)

* *p* < 0.05 (comparison with patients who reach or exceed the cut‐off, within the same phenotype).

**FIGURE 2:**
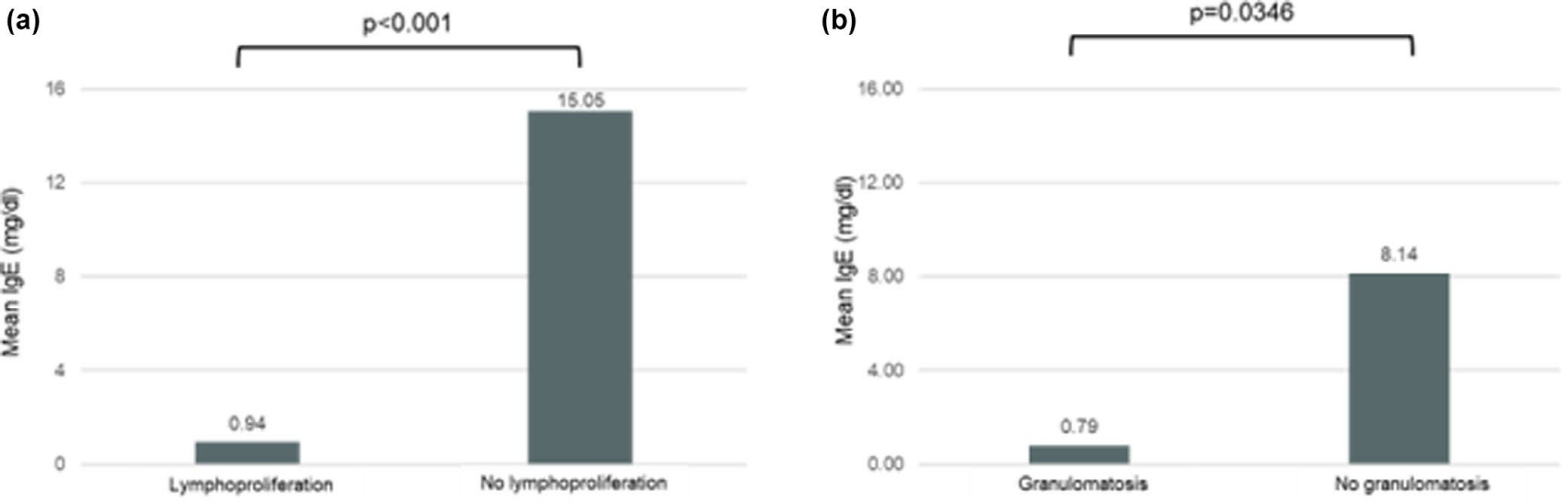
Mean serum IgE levels in CVID patients with lymphoproliferation (a) and granulomatosis (b).

**FIGURE 3:**
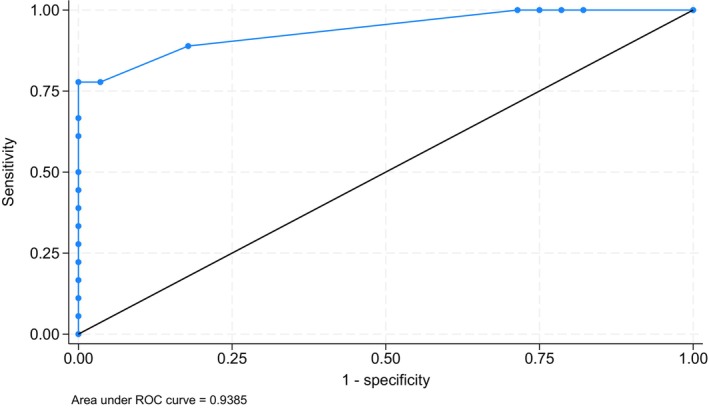
ROC analysis of IgE in CVID patients with lymphoproliferative phenotype vs. non‐lymphoproliferative one.

Patients with the lymphoproliferation phenotype exhibited statistically significant lower IgE values compared with non‐lymphoproliferation phenotypes (−14.1 kU/L, 95% CI: −8.0 to −20.2, *p* < 0.001, power = 94%) (Figure 2a). According to the ROC analysis (Figure 3), the area under the curve (AUC) was 94% for the total IgE levels to predict lymphoproliferation (SE 0.036, 95% CI: 87% to 100%). Our calculated best cut‐off value was total IgE levels = 2.4 kU/L, showing 100% sensitivity and 67% specificity. The positive predictive value was 82%, whereas the negative predictive value was 100%.

**FIGURE 4:**
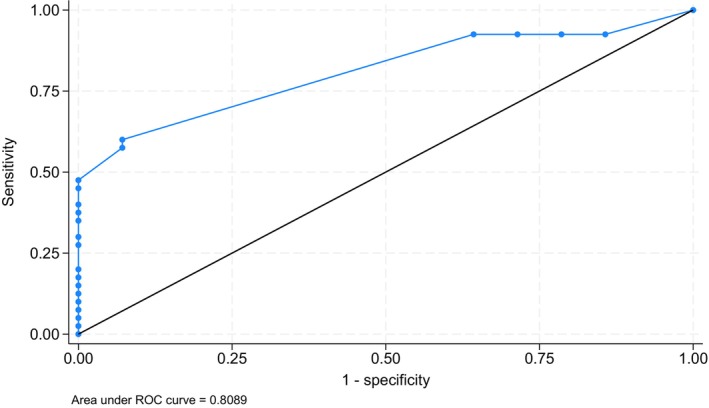
ROC analysis of IgE in CVID patients with granulomatous phenotype vs. non‐granulomatous one.

Patients with granulomatous disease exhibited statistically significant lower serum IgE values, compared with patients without granulomatosis (−7.3 kU/L, 95% CI: 0.5 to 14.1, *p* = 0.035, power = 95%, Figure 2b). Furthermore, all the patients with granulomatous phenotype had serum IgE < 2.5 kU/L (*p* = 0.005) (Table 5). The ROC analysis (Figure 4) showed an AUC of 81% for total IgE levels to predict the granulomatous phenotype (SE 0.053, 95% CI: 70% to 91%). The sensitivity and specificity were 93% and 60%, respectively, at our best cut‐off of total IgE = 1.8 kU/L. The positive predictive value was 45%, while the negative predictive value was 96%.

Conversely, we detected only slight and non‐significant differences in IgE values in patients with the other phenotypes, compared with the total study population. characterized by malignancies (−2.1 kU/L, 95% CI: 4.0 to −8.3, *p* = 0.486, power = 11%), autoimmunity (−1.8 kU/L, 95% CI: 5.2 to −8.7, *p* = 0.607, power = 8%), recurrent respiratory infections (+1.8 kU/L, 95% CI: 5.2 to −8.7, *p* = 0.607, power = 8%), or enteropathy (−5.0 kU/L, 95% CI: 3.2 to −13.1, *p* = 0.227, power = 66%). Additionally, there was no significant correlation between these phenotypes and the IgE < 2.5 kU/L cut‐off.

### 3.5 Serum IgA Levels According to Clinical Phenotypes in CVID Patients

We documented statistically significant lower IgA serum values in patients with clinical phenotypes driven by lymphoproliferation (−22.4 kU/L, 95% CI: 14.2 to 30.7, *p* < 0.001, power = 100%), granulomatous disease (−14.7 kU/L, 95% CI: −3.7 to −25.8, *p* = 0.010, power = 97%), and recurrent respiratory infections (−12.0 kU/L, 95% CI: −0.7 to −23.3, *p* = 0.037, power = 58%) compared with patients without these phenotypes, respectively. The lymphoproliferative phenotype was more frequently associated with IgA < 7 mg/dL (78% of them had serum IgA < 7 mg/dL, *p* < 0.001). The same results were observed in patients with granulomatous disease (85% of them with serum IgA < 7 mg/dL, *p* = 0.007).

**FIGURE 5:**
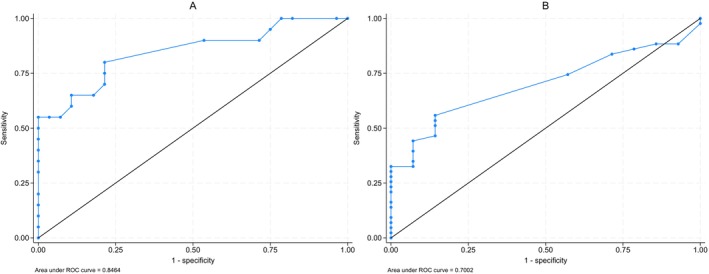
ROC analysis of serum IgA levels in CVID patients with lymphoproliferative phenotype vs. non‐lymphoproliferative one (A) and in CVID patients with granulomatous vs. non‐granulomatous disease (B).

The AUC of the IgA serum values to predict the lymphoproliferative and granulomatous phenotypes was 85% (SE 0.060, 95% CI: 73% to 96%) and 70% (SE 0.070, 95% CI: 56% to 83%), respectively (Figure 5). However, the diagnostic accuracy of IgA in predicting these phenotypes was lower than IgE.

On the other hand, patients with enteropathy did not show significantly lower serum IgA values compared with the other patients without enteropathy (−11.7 kU/L, 95% CI: 1.6 to −25.0, *p* < 0.083, power = 43%). There were no statistically significant differences observed for autoimmunity (+1.4 kU/L, 95% CI: 12.2 to −9.4, *p* = 0.794, power = 6%), or malignancies (+4.5 kU/L, 95% CI: 14.5 to −5.5, *p* = 0.367, power = 14%) either.

## 4 Discussion

CVID is the most common symptomatic primary antibody defect in adulthood, characterized by significantly reduced serum immunoglobulin levels and a diminished or absent capacity to produce specific antibodies.

CVID involves a complex interplay of genetic, epigenetic, and environmental factors. The resulting imbalance of the immune system makes patients especially vulnerable to recurrent upper and lower respiratory tract infections, autoimmune diseases, malignancies, and lymphoproliferative disorders. Chronic lung disease is a common issue among CVID patients, leading to repeated hospitalizations and significant morbidity [6, 16, 17, 18]. Respiratory symptoms may stem from recurrent infections and scarring lung changes [17]. Equally, when diagnosing patients with recurrent sinopulmonary and cutaneous infections, it is crucial to consider not only primary or secondary immunodeficiencies but also anatomical abnormalities and underlying atopy. The clinical manifestations of infections in CVID patients, such as bronchiectasis and non‐allergic rhinosinusitis, can overlap with allergic conditions, complicating the recognition of allergy in CVID presentations [19].

Epidemiological studies report a prevalence of approximately 4.4% for asthma [20] and up to 50% in some countries for rhinitis [21], in the general population. Prevalence of allergic diseases in CVID patients varies widely across studies, ranging from 12% up to 42%. Most cohorts report allergic rhinitis and asthma as the predominant manifestations. This heterogeneity of prevalence likely reflects differences in cohort selection, allergy assessment methods, and diagnostic definitions across studies (e.g., allergic‐ and asthma‐like symptoms were identified through retrospective chart review and patient‐reported data across several centres, which may have overestimated true allergic disease). In our cohort, the prevalence (26.6%) falls in the mid‐range of published estimates and is consistent with studies using structured allergy assessment, as it was done in our study. Moreover, these findings may reflect differences in ascertainment and definitions rather than true epidemiological discrepancies. Therefore, the lower prevalence of allergic rhinitis and asthma compared with previous studies may be explained using objective diagnostic methods (skin testing, sIgE levels, allergy‐focused medical history), rather than relying solely on self‐reported symptoms, as done in most previous reports. However, the allergy‐related findings are based on a relatively small number of confirmed IgE‐mediated cases and should therefore be interpreted primarily as descriptive rather than definitive prevalence estimates.

These differences may reflect that previous studies and the present cohort are capturing related but not identical clinical entities. In CVID, respiratory and allergic‐like manifestations may arise from heterogeneous mechanisms, including true IgE‐mediated allergy, infection‐related inflammation, and immune dysregulation–driven airway disease, which may have been variably classified across cohorts.

In 2020, Kotsiou et al. [22] underscored the importance of early respiratory surveillance in CVID patients, given the high prevalence of respiratory complications. Furthermore, the coexistence of CVID and asthma can pose challenges for early diagnosis, as CVID can mask or mimic refractory asthma [22], emphasizing the need for comprehensive clinical evaluation and timely interventions to enhance management strategies and patients' quality of life [5, 23]. Fekrvand et al. [24] stressed the need for broader management approaches for CVID patients with severe, treatment‐resistant asthma, whereas Ibrahim et al. [25] highlighted the importance of evaluating alternative diagnoses in asthma patients unresponsive to therapy, recommending serum immunoglobulin testing.

Although CVID manifestations are well described, the relationship between CVID and allergy is less understood. Allergic diseases have long been observed among IEI patients, particularly those with Primary Antibody Deficiency [19]. It has been suggested that allergic diseases may be a characteristic CVID clinical phenotype, even at the disease's onset [3]. The association between allergy and IEI reflects an imbalance in immune regulation, potentially influenced by microbial colonization and infection patterns [5, 26].

Hartman et al. [27] emphasize the role of skin testing and oral challenges for beta‐lactam hypersensitivity in CVID patients, improving infection management and reducing costs. Bjelac et al. [28] recommend allergy testing for suspected allergic diseases in CVID patients due to the unpredictability of hypersensitivity responses.

In our series of 60 CVID patients, the prevalence of allergies and DHRs was 26.6%, as in the literature, being allergic rhinitis (9/16, 56.2%) and asthma (2/16, 12.5%) the most common manifestations in the 16 CVID patients with allergies and/or hypersensitivity reactions. No clinical differences were observed between onset in childhood and adulthood [29]. Notably, in our cohort, no cases of latex allergy, hymenoptera venom allergy, or food allergy were observed. In addition, it should be emphasized that data regarding DHRs must be interpreted with caution, as it is not always possible to confirm an IgE‐mediated mechanism. In fact, the prevalence of allergies excluding DHRs was 16.6%.

CVID patients have constitutive reductions in serum IgG and IgA, with or without low IgM, but even lower total IgE levels than the general population [11]. Various cut‐off points, such as levels below 2 kU/L or 2.5 kU/L, have been proposed to define IgE‐deficiency [10]. Despite common beliefs that low IgE levels are inconsequential, recent studies suggest potential links between low IgE levels and primary immunodeficiencies [11].

To evaluate the role of low IgE producers, we measured patients' total IgE levels. In our investigation, we found higher IgE levels in CVID patients with respiratory allergies compared with those without. Not surprisingly, the higher IgE values were specific to patients with a pollen and dust mites allergy compared with patients with DHRs, as these last are frequently non‐IgE‐mediated reactions. Moreover, as already described with repeated booster vaccination [22], it is possible that CVID patients can switch and produce IgE more efficiently if there is a persistent antigen immune stimulation, such as a perennial or seasonal allergen. However, although higher than the allergy‐free patients, the total IgE values of allergic CVID patients remain under the normal range of local laboratories.

For completeness, two patients with allergic‐like manifestations showed negative skin prick test results and IgE < 2 kU/L. Of them, one patient complained of allergic rhinitis, while another had asthma and rhinitis. More often, patients with CVID have a clinical history that only suggests an allergic respiratory disease. Assessing asthma and atopy in CVID patients remains challenging due to distinguishing whether respiratory symptoms stem from infectious complications or allergic disease [5, 30]. Importantly, respiratory symptoms suggestive of allergy in CVID may not align with classical IgE‐mediated atopy, reflecting the underlying immune dysregulation characteristic of this condition. Therefore, our results are more appropriately framed as describing “allergic and allergic‐like manifestations” within CVID, rather than as strict epidemiological comparisons with the general population.

Some studies have reported altered cytokine milieus in CVID, including increased IL‐4 levels in a subset of patients [31]. However, this finding has not been consistently replicated. Hel [32] observed reduced Th1 (IFN‐γ, IL‐2), Th2 (IL‐9, IL‐13), and Th17 (IL‐17) cytokines in CVID compared with healthy donors, while Kutukculer [33] described decreased absolute numbers of CD4^+^IL‐4^+^ T cells and no significant alterations in T‐reg populations. Varzaneh et al. [34] further documented dysregulation of several cytokines, including IL‐4, IL‐5, IL‐10, and IL‐12, although cytokine data across studies remain inconsistent. Regarding T‐reg cells, some authors have reported reduced frequencies [35] or qualitative and quantitative defects [36]. Collectively, these findings might suggest that immune dysregulation—rather than classical IgE‐mediated mechanisms—may contribute to certain allergic‐like manifestations in CVID, although no study to date has demonstrated a direct mechanistic link.

It is known in the literature that patients with Selective IgA Deficiency (SIgAD), the most common primary humoral immunodeficiency, have a higher risk of developing allergic diseases, with a prevalence of up to 84% [37]. Allergic CVID patients in our study had higher average IgE levels than in CVID patients without allergies, although their levels remain below the normal range of the healthy population. However, CVID patients with IgA < 7 mg/dL and allergic manifestations did not show an IgE increment. Many studies investigated the pathogenetic link behind CVID and SIgAD, but the exact mechanisms are not fully understood [38]. It is not unlikely that patients with SIgAD can produce more IgE in the presence of allergies compared with CVID allergic patients with absolute IgA deficiency (serum IgA levels < 7 mg/dL). New studies are required to clarify the pathogenetic mechanisms behind these observations.

Considering the significant correlation between serum IgE and IgA levels, we propose low or undetectable IgE levels could support the diagnosis of CVID and humoral similar conditions [11].

However, very low or undetectable serum IgE should not be interpreted as a disease‐specific marker of CVID nor simply as a surrogate for the absence of atopy. Population‐based studies have shown that ultra‐low IgE levels (< 2–2.5 kU/L) are uncommon in the general population and are associated with a broader phenotype of immune dysregulation, including inborn errors of immunity, autoimmunity and malignancy [7, 8, 9, 10, 11]. Importantly, patients with immune deficiencies may present allergic or allergic‐like manifestations despite undetectable IgE levels ^10,11;19^. Rather than representing a benign immunological variant, IgE deficiency may reflect impaired immune homeostasis characterized by cytokine imbalance and regulatory defects [32, 33, 34, 35, 36]. Within this framework, CVID may represent one clinical manifestation along a wider spectrum of immune dysregulation associated with the “very‐low IgE producer” phenotype rather than the defining context for this biomarker [8, 10, 11]. Accordingly, the associations observed in our cohort should be interpreted as supporting a contextual biomarker of disease severity and immune dysregulation rather than a validated prognostic or stratification tool specific to CVID.

Diagnostic process for patients with recurrent infections and suspected hypogammaglobulinemia omits the assessment of total IgE. Routine measurement of serum total IgE could aid in distinguishing CVID from other conditions with similar symptoms, improving the timeliness of diagnosis and initiation of immunoglobulin replacement therapy (IgRT) which can reduce CVID‐associated morbidity and mortality [39].

Certainly, incidentally discovered IgE deficiency warrants further investigation for symptoms suggestive of immunodeficiency. A recent study indicates a high prevalence (74%) of undetectable IgE in CVID patients, potentially linked to a relative decrease in IgG4 and isotype‐switching defects [11]. These findings reveal that a low serum IgE, assumed to indicate atopy absence, is infrequent in the general population (3.3%) [8, 11]. This data suggests that in CVID patients meeting diagnostic criteria, an IgE < 2 kU/L enhances diagnostic certainty, while a high IgE (>180 kU/L) warrants alternative diagnoses [11]. Contrastingly, an IgE < 2 kU/L is unusual for secondary hypogammaglobulinemia due to immunosuppressants or protein loss, occurring in only 8.8% of patients [11].

CVID's clinical manifestations include increased malignancy susceptibility. Epidemiological studies in ‘allergo‐oncology’ suggest an inverse association between allergic diseases and malignancies [8], hypothesizing an IgE role in antitumor surveillance. A recent study demonstrated how malignancy incidences were higher in IgE‐deficient patients compared with non‐deficient controls [10]. Agress et al. found malignancy diagnosis in 23.5% of IgE‐deficient patients [14]. In our investigation, the relationship between cancer and IgE is not statistically significant, and nearly 38% of patients with a malignancy history had IgE deficiency, though the sample size limited statistical significance (power = 11%).

We found a relationship between clinical phenotypes and serum total IgE levels. In our 60 CVID patients, serum IgE < 2.5 kU/L values significantly correlate with the lymphoproliferative phenotype, characterized by persistent lymphadenopathy, hepato‐splenomegaly, and/or granulomatous disease. In addition, a separate analysis revealed a statistically significant correlation between serum IgE < 2.5 kU/L and granulomatous disease, known for its severity [40]. Regarding the other CVID clinical characteristics, there was no significant correlation between serum IgE < 2.5 kU/L and clinical phenotypes associated with recurrent infections, malignancy, enteropathy, and autoimmunity manifestations. However, the limited sample size makes the study not well‐powered for these analyses, and the results should be confirmed by larger studies. Considering these significant correlations with severe clinical phenotype, we can suggest total IgE's potential prognostic role, indicate increased morbidity and mortality, and warrant close monitoring. Although low IgE strongly correlated with lymphoproliferation and granulomatous disease, its role reflects immune dysregulation rather than simply absence of atopy [10].

Regarding other immunoglobulin classes, we found that serum IgA < 7 mg/dL significantly correlates with the lymphoproliferative phenotype. However, it is worth noting that IgA diagnostic accuracy is lower than IgE in predicting this phenotype, and total IgE has greater sensitivity and specificity. The IgE and IgA correlation, using both in combination, increases total prognostic accuracy.

For infectious complications, it is well known that CVID patients have airways chronic inflammation and recurrent respiratory infections [17]. Our data show a statistically significant correlation between recurrent respiratory infections and serum IgA < 7 mg/dL, but no correlation with IgE levels. Mucosal immunity alterations likely contribute to infections, highlighting the different and complementary prognostic roles of IgA and IgE. Further research is essential to deepen our understanding of IgE and IgA's prognostic role in CVID patients.

## 5 Conclusion

This study sheds light on the complex relationship between CVID, allergic manifestations, and immunoglobulin serum levels, particularly IgE and IgA. Our findings confirm that allergic diseases are prevalent among CVID patients, with allergic rhinitis being the most common. Despite lower IgE levels in CVID patients compared with the general population, those with respiratory allergies exhibited relatively higher IgE values, highlighting the interplay between immune dysregulation and allergen exposure.

Significant correlations were observed between IgE levels < 2.5 kU/L and severe CVID phenotypes, such as lymphoproliferative disorders and granulomatous disease, suggesting serum IgE as a prognostic marker for CVID morbidity and mortality. Combined IgE and IgA assessments improved diagnostic accuracy, emphasizing their complementary value.

The study analysed IgE and IgA levels in a robust cohort of CVID patients, providing insights into their diagnostic and prognostic roles. By exploring allergic manifestations in CVID patients, it contributed valuable data on the prevalence and characteristics of allergies in this population that is lower compared with the previous studies, as we adopted objective diagnostic tools (skin test, sIgE levels, and allergy‐focused medical history) rather than exclusive reliance on self‐reported symptoms, as commonly reported in earlier studies.

However, subgroup analyses lacked statistical power, and the absence of longitudinal follow‐up limited causality assessments. In addition, IgE could be interpreted as a biomarker of immune dysregulation and disease severity, rather than as a validated prognostic or stratification tool specific to CVID. Further research is needed to clarify the relationships between CVID with allergies, IgE‐deficiency, and allergic SIgAD patients. In conclusion, IgE and IgA measurements should be integral to CVID evaluation, potentially improving early detection and management of severe phenotypes.

## Funding

The authors have nothing to report.

## Conflicts of Interest

The authors declare no conflicts of interest.

## Supporting information


**Data S1:** Supporting Information.

## Data Availability

The data that support the findings of this study are available on request from the corresponding author. The data are not publicly available due to privacy or ethical restrictions.
