# Supplementary material for: Allergic Diseases in Common Variable Immunodeficiency: A Prospective Cross‐Sectional Study on Prevalence and Allergy Biomarkers of Clinical Phenotypes
Source: Scand J Immunol. 2026 Mar 17;103(3):e70108. doi: 10.1111/sji.70108 (PMC12996437; doi:10.1111/sji.70108)
Supplement: Supplementary file 1 — Data S1: Supporting Information. [file SJI-103-e70108-s001.docx]

**Supplementary Materials**

**Methods and Bibliographic research**

1. **Methods**

***1.1 Design and Patients***

This prospective, cross-sectional, case-control study included adult patients, affected by CVID, who were diagnosed and followed up at the Immunologia delle Malattie Rare e dei Trapianti, Clinica Medica, from Azienda Ospedaliero Universitaria delle Marche and Università Politecnica delle Marche in Ancona (Central Italy). Our Centre is the referral one in Marche Region for IEI in adulthood, is included in the IPINet Italian registry for CVID and is “Documenting Center” for ESID (https://esid.org/Working-Parties/Registry-Working-Party/Documenting-centers).

Patients included in this study were ≥18-year-old and were diagnosed with CVID based on the revised criteria established by the ESID^1^. We excluded patients diagnosed with one of the Primary Immune Regulatory Disorders, such as Autoimmune Lymphoproliferative Syndrome and CTLA-4 deficiency.

We assessed the presence of allergic diseases by studying individuals at the Allergology Unit of the same University Hospital. The allergy assessment included allergic rhinitis, bronchial asthma, food allergies, Hymenoptera venom allergy, latex allergy, and drug hypersensitivity. Concerning drug hypersensitivity, we have considered type B hypersensitivity and excluded type A reactions ^26^.

Patients who died during follow-up were also comprehended in the study, considering the causes and dates of death. All patients enrolled in the study provided informed consent for the collection and publication of their data (Prot. n: 2016 0561 OR del 27/10/2016; DG n. 871 7/12/2016). The study was conducted following the principles of Good Clinical Practice, the guidelines of the International Conference on Harmonization, and the Declaration of Helsinki, which delineate ethical standards for medical research involving human subjects.

***1.2 Data Collection and Evaluation***

Comprehensive data collection was conducted for each patient, including demographic information and medical history. This included details such as date of birth and death (reported cause of death), age of symptom onset, clinical manifestations at diagnosis and during follow-up, comorbidities, concurrent medications, family history of immunodeficiencies, allergic and autoimmune diseases, consanguinity among parents and grandparents, as well as history of malignancy and associated therapies.

Our patients underwent regular clinical follow-up appointments every 6 months or when clinically indicated. This follow-up consisted of a physical examination and a detailed medical history, with specific attention to recurrent infections and the development or progression of disease complications.

Routine laboratory tests for CVID diagnosis were performed at diagnosis and during follow-up, according to what already published ^13,14^.

The following tests/procedures were performed for the diagnosis of allergic conditions:

- Physical examination and medical history to collect clinical manifestations of allergic diseases,
- Measurement of peripheral blood eosinophils,
- Skin testing,
- Measurement of total and specific IgE,
- Lung function testing (asthma diagnosis as per GINA criteria^15^).

Additionally, we delineated the distribution of CVID-associated complications according to the categorization outlined by Chapel et al ^3^.

Diagnostic delay was defined as the time between the occurrence of the first CVID-related symptom (e.g. infection recurrences, ITP, neoplasia) and the effective CVID diagnosis. Therapeutic delay was defined as the time between the occurrence of the first CVID-related symptom and the initiation of Ig replacement therapy.

All patients received immunoglobulin replacement therapy with subcutaneous immunoglobulin (SCIg) or intravenous immunoglobulin (IVIg). Infective episodes and serum IgG levels (target serum IgG levels around 700-800 mg/dl) determined the dose adjustments. For those patients who did not respond adequately to immunoglobulin treatment, antibiotic prophylaxis was administered. In cases of autoimmune complications, treatment strategies involved the use of glucocorticoids, immunosuppressants, and biological drugs^14^.

***1.3 Statistical Analysis***

The primary analysis investigated the total IgE (absolute values and frequency of patients with IgE < 2.5 kU/L) in the study population and the prevalence of allergic diseases, compared to the general population. In the secondary analyses, we investigated the relationships between IgE and: I) the clinical phenotypes of interest; II) other Ig classes (absolute values and frequency of patients with IgA < 7 mg/dl). In addition, correlations between IgA and the clinical phenotypes were analysed to compare the diagnostic/prognostic accuracy between IgE and IgA.

Categorical variables were presented as absolute (n) and relative frequencies (%), while average values of continuous variables were reported as means (± standard deviation, SD) or medians (with interquartile ranges, IQR), as appropriate.

Quantitative comparison of IgE values was conducted using the one-way ANOVA test, and the chi-squared test was employed for comparing IgE categories between groups. Spearman’s correlation test was utilized to investigate the correlations between Ig classes.

Receiver operating characteristic (ROC) curves were generated to assess the accuracy of IgE values in detecting the CVID phenotypes of interest. Area Under the Curve (AUC) was calculated, along with sensitivity and specificity at the best cut-off values determined using the Youden index (i.e., highest sum of sensitivity and specificity).

A post-hoc power calculation was performed for the obtained statistically significant differences (alpha=5%). The statistical analyses were conducted using STATA v.18 (StataCorp - College Station, Texas, USA).

1. **Bibliographic research**

A research study was conducted by searching PubMed using the MeSH terms “Common Variable Immunodeficiency” combined with either “Hypersensitivity” or “IgE” to assess whether previous research had studied this relationship. Studies only in English language from 2014, January 1st, to 2025, March 31st, were considered according to the PRISMA guidelines ^17^. We did not register this (systematic) review in PROSPERO or Open Science Framework (OSF) repository ([www.crd.york.ac.uk/prospero/](http://www.crd.york.ac.uk/prospero/)).

The flowchart describes the research studies identified in our review via database.

**Identification of studies via databases**

Records identified through databases searching

(n = 35)

Additional records identified through other sources

(n = 0)

**Identification**

Records after duplicates removed.

(n = 4)

**Screening**

Records screened.

(n = 31)

Full-text articles excluded (n = 19):

By title (n =11)

By full text (n = 8)

**Eligibility**

Full-text articles assessed for eligibility.

(n = 31)

Studies included in qualitative synthesis.

(n = 12)

Subsequent analysis deemed 12 of the 35 studies eligible. We excluded duplicate papers (n = 4) and those whose subsequent analysis of the text, title, and abstract indicated they did not meet thematic inclusion criteria (n=19).

Table 1S. Studies in the literature review using the MeSH terms “Common Variable Immunodeficiency” combined with either “Hypersensitivity” or “IgE”.

| Author [ref.no] | Type of article | Title | Aim of the article | Note |
| --- | --- | --- | --- | --- |
| Imam K et al, 2024[⁷] | Review | Isotype deficiencies (IgG subclass and selective IgA, IgM, IgE deficiencies). | The selective isotype deficiencies have a variable presentation from asymptomatic to severe recurrent infections. | IgE deficiency is associated with reduced response to vaccination and an increased risk of malignancy, particularly in patients with no allergic manifestations. The authors strongly suggested strict monitoring for malignancy in patients with selective IgE deficiency. |
| Agress et al., 2024 [^8^] | Retrospective analysis in 408 adults with serum IgE deficiency | The Association Between Malignancy, Immunodeficiency, and Atopy in IgE-Deficient Patients. | To examine the association between malignancy and atopy or other immune abnormalities, in patients with IgE deficiency. | Agress et al. have identified a malignancy in 96 out of 408 (23.5%) IgE-deficient patients. However, the risk of malignancy was not uniformly distributed among all IgE-deficient individuals. Patients with both an IgE deficiency and another non-CVID humoral abnormality (such as low levels of IgG, IgA, or IgM without meeting the criteria for CVID) were more likely to be diagnosed with malignancy compared to those with only a selective IgE deficiency. This risk is higher in patients with IgM or IgG2 deficiency and in those with CD4 lymphopenia. |
| Yıldız et al., 2023 [^21^] | Retrospective study in 84 adult patients with CVID | [Allergic Diseases as a Clinical Phenotype Marker in Patients with Common Variable Immunodeficiency](https://pubmed.ncbi.nlm.nih.gov/37473738/) | To evaluate the prevalence and clinical and laboratory characteristics of different allergic diseases in patients with CVID. | Authors documented atopic dermatitis, drug hypersensitivity reaction, allergic rhinitis and asthma in 2.4, 6, 8.3 and 25%, respectively, adult CVID patients. Serum IgE levels, CD19+ B cell, switched memory B cell, and Natural killer cells were higher in CVID patients with allergic disease. Allergic diseases should be considered as markers of the clinical phenotype in CVID. |
| Kilinc M et al. 2023 [^9^] | Retrospective cohort study in 62 adult patients with CVID between April 2012 and December 2021 | Relationship between autoimmune diseases and serum basal immunoglobulin E levels in patients with common variable immunodeficiency | To explore the relationship between autoimmunity and low IgE levels in adult patients with CVID. | In this series of patients with CVID, 23/ 62 (37%) had at least one autoimmune disease, with most of them having very low serum IgE levels (<2.5 IU/mL).  The median (interquartile range) serum IgE value in these patients was significantly lower as compared to that of patients with CVID without autoimmune disease (p < 0.001). Low IgE levels were an independent risk factor for the occurrence of autoimmune disease in patients with CVID (odds ratio 3.081 [95% confidence interval, 1.222-7.771]; p = 0.017). |
| Matricardi PM. 2023 [^10^] | Review | The Very Low IgE Producer: Allergology, Genetics, Immunodeficiencies, and Oncology. | To revise the available data on very low IgE producers. | Low IgE producers have been associated with common variable immunodeficiency, lung diseases, and cancer. There have been a few epidemiological studies that have documented a higher cancer risk among very low IgE producers. This has led to a controversial hypothesis that proposes a new and evolutionary-relevant role for IgE antibodies in antitumor immune surveillance. |
| Ibrahim et al., 2022 [^24^] | Single Case report | [Recurrent asthma exacerbations: co-existing asthma and common variable immunodeficiency](https://pubmed.ncbi.nlm.nih.gov/33902374/) | This paper presents the case of a 43-year-old woman with a long history of recurrent wheezing, coughing, sinusitis, and multiple lower respiratory tract infections. Antibiotics and steroids only temporarily alleviated these episodes, which had been occurring for many years. She had a longstanding clinical diagnosis of asthma. | Further tests for her recurrent infections revealed low serum IgG, IgA, and IgM levels consisting of the diagnosis of CVID. In patients with asthma who do not respond to conventional therapy, it is crucial to search for alternate or co-existing diagnoses. Measuring serum immunoglobulin levels should be included in this comprehensive evaluation. |
| Rubin et al., 2022 [^4^] | Multicentre prospective study in 79 CVID patients | [Allergic-like disorders and asthma in patients with common variable immunodeficiency: a multi-center experience](https://pubmed.ncbi.nlm.nih.gov/33297810/) | In this study, authors investigated potential associations between CVID and allergies. | Authors documented an allergic-like disorder in 65% of CVID patients having non-elevated serum IgE levels. Allergic CVID patients had a higher rate of bronchiectasis on chest CT. The authors suggest that timely diagnosis and stratification of allergy in CVID patients can improve their outcome and quality of life and promote appropriate treatment and better management of pulmonary exacerbations. |
| Fekrvand S. et al.2020 [^23^] | Letter to the Editor | [Are asthma and allergic diseases phenotypic markers for patients with common variable immunodeficiency?](https://pubmed.ncbi.nlm.nih.gov/32217189/) | To determine the functional and structural changes in the respiratory tract of patients with CVID following recurrent infections. It also acknowledges the relevance of allergic disorders such as asthma, atopic dermatitis, eczema, food allergy, allergic rhinitis, and conjunctivitis in CVID patients. | The study concluded that the prevalence of asthma in CVID patients was lower (2.8%) compared to some higher reported rates. The authors suggest considering asthma and other allergic disorders as phenotypic markers for a subgroup of CVID patients. Moreover, the study detected a slightly higher rate of mortality in patients with asthma as compared to the whole CVID series (28.5 vs 27.3%). |
| Bjelac et al., 2018 [^26^] | Retrospective medical record review of 160 CVID patients | [Allergic disease in patients with common variable immunodeficiency at a tertiary care referral center](https://pubmed.ncbi.nlm.nih.gov/29273136/) | To explore the prevalence of allergic diseases in patients with CVID at the authors' institution. Additionally, it aimed to identify demographic and laboratory characteristics that facilitate the identification of patients with CVID who are more likely to have IgE-mediated comorbid diseases. | Authors detected a history consistent with asthma, allergic rhinitis, penicillin allergy, and food allergy in 37.5, 55.6, 21.2, and 11.2% of medical records, respectively. Moreover, 10% of asthma and 11% of rhinitis patients had positive specific IgE results, while only two patients showed positive specific IgE for food allergens. These findings highlight the importance of conducting allergy testing in all CVID patients with a clinical history suggestive of allergic disease. |
| Lawrence et al., 2018 [^11^] | Multicentre cohort study in 354 children and adult with CVID | [Low Serum IgE Is a Sensitive and Specific Marker for Common Variable Immunodeficiency (CVID)](https://pubmed.ncbi.nlm.nih.gov/29453744/). | To evaluate whether low/undetectable serum IgE is characteristic of CVID by comparing the frequency of low/undetectable serum IgE in healthy controls and patients with CVID. | Authors documented undetectable serum IgE levels (<2 IU/ml) in 75.6% (95% CI, 65.6–85.7%) of patients with CVID as compared to 3.3% of the general population. High serum IgE levels (>180 IU/ml) were rare in CVID (0.3% of patients). Allergen specific IgE was not detectable in 96.5% of patients with CVID. IgG1/IgG4 ratio was increased in subjects with low IgE, regardless of whether they are controls or have CVID. Very low IgE levels are indicative of a primary humoral immunodeficiency. These results support for the regular assessment of serum IgE levels in the work-up of patients with hypogammaglobulinemia. |
| Yazdani et al., 2016  [^5^] | Retrospective analysis in 187 children with CVID | Asthma and Allergic Diseases in a Selected Group of Patients with Common Variable Immunodeficiency | To report the prevalence of asthma and allergic diseases in patients with common variable immunodeficiency (CVID) and to evaluate the association between these conditions and clinical and immunological conditions. | The study found that asthma and allergic diseases were present in 22/187 (11,8%) CVID patients, with a higher IgE and lower IgM serum levels observed in those with these conditions. Authors documented asthma, atopic dermatitis, allergic eczema, food allergy, and urticaria in 4.8, 3.2, 2.1, 2.6, and 2.1% of patients, respectively. These findings suggest that immune dysregulation and skewed Ig class switching towards IgE may contribute to atopic diseases in CVID patients. |
| Hartman et al., 2017 [^25^] | Letter to the Editor regarding 100 patients | [Lack of Clinical Hypersensitivity to Penicillin Antibiotics in Common Variable Immunodeficiency](https://pubmed.ncbi.nlm.nih.gov/27873106/). | To determine how many people with CVID report being allergic to beta-lactam antibiotics and to verify these allergies through skin testing and controlled dose challenges performed in a clinical setting. | Patients with CVID who have a history of beta-lactam hypersensitivity should optimally undergo skin testing and oral challenges in a clinical setting. The institution of this objective testing could improve the management of infections and decrease the economic costs associated with antibiotic usage in patients with CVID. |
